# Identification of *Pseudomonas* strains for the biological control of soybean red crown root rot

**DOI:** 10.1038/s41598-022-18905-2

**Published:** 2022-08-25

**Authors:** Khin Thuzar Win, Michie Kobayashi, Fukuyo Tanaka, Kasumi Takeuchi, Aung Zaw Oo, Chang-Jie Jiang

**Affiliations:** 1 Institute of Agrobiological Sciences, National Agriculture and Food Research Organization (NARO), Tsukuba, 305-8602 Japan; 2 Research Center for Advanced Analysis, National Agriculture and Food Research Organization, Tsukuba, Ibaraki Japan; 3 Japan International Research Center for Agricultural Sciences, 1-1 Ohwashi, Tsukuba, Ibaraki 3058686 Japan; 4 Rice Research Institute, Shandong Academy of Agricultural Sciences, Jinan, 250100 China

**Keywords:** Microbe, Biotic, Applied microbiology

## Abstract

Soybean red crown root rot (RCR), caused by the soil-borne fungal pathogen, *Calonectria ilicicola*, is the most destructive disease affecting soybean production in Japan. To date, no resistant cultivars or effective fungicides have been developed to control this disease. In this study, we evaluated 13 bacterial strains to determine their efficacy in controlling *C. ilicicola*. We first investigated whether the volatile organic compounds (VOCs) emitted by the bacterial strains exhibited any antifungal activity against *C. ilicicola* using the double-plate chamber method. The results showed that VOCs from three *Pseudomonas* bacterial strains, OFT2 (*Pseudomonas* sp.), OFT5 (*Pseudomonas* sp.), and Cab57 (*Pseudomonas protegens*), exhibited strong inhibitory activity against *C. ilicicola* mycelial growth. Some antifungal activity was also observed in the culture supernatants of these *Pseudomonas* strains. Greenhouse soil inoculation tests showed that application of OFT2, OFT5, and Cab57 cultures around soybean seeds after seed sowing significantly reduced the severity of RCR, as shown by up to 40% reduction in *C. ilicicola* fungal growth in the roots and 180–200% increase in shoot and root fresh weights compared to the water control. Our results suggest that OFT2, Cab57, and OFT5 produce potent antifungal compounds against *C. ilicicola*, thereby showing considerable potential for the biological control of *C. ilicicola* during soybean production.

## Introduction

Soybean red crown root rot (RCR), caused by the soil-borne fungal pathogen, *Calonectria ilicicola*, is a destructive disease observed in soybean fields worldwide. The disease is characterized by root rot, damping-off of young seedlings, and early defoliation^1,2^. It occurs mostly when seeds are grown in poorly drained soils with high clay content and/or when fields are subjected to temporary flooding and ponding. The claying nature of such soybean fields is favorable for paddy cultivation, making them prone to short-term waterlogging owing to poor soil drainage^3^. Soybean RCR is a major limiting factor responsible for low soybean grain yield in Japan, as more than 80% of soybean crops are grown in paddy-converted fields in Japan^4^. Soybean yield losses due to RCR are estimated to range from 25 to 30%^5,6^ to as high as 50% depending on the environmental conditions that favor fungal colonization^2^.

Soil-borne pathogens pose a great challenge in crop production as most of them are very difficult to control via conventional agronomic practices, such as the use of resistant cultivars and synthetic fungicides and crop rotation^1,7,8^. The number of resistant plant varieties is limited, and the delivery of fungicides around the roots is inefficient, especially during the late stages of plant growth. In addition, excessive use of synthetic fungicides can result in the emergence of new pathogen isolates with fungicide resistance^9^. As with most other soil-borne pathogens^7,8^, *C. ilicicola* is very difficult to control due to its wide host range and longevity in soil and/or plant residues^10^. *C. ilicicola* infects 15 plant species^11^ and can survives as microsclerotia in the soil for at least 7 years under natural conditions^12,13^. Currently, no resistant soybean cultivars or effective fungicides are available for the control of *C. ilicicola*^10^.

Biological control using beneficial microorganisms is receiving scientific and commercial attention as a promising alternative or a supplemental strategy for the management of soil-borne pathogens^9,14^. Many microbial strains, including *Trichoderma* fungi as well as *Bacillus* and *Pseudomonas* bacteria, have been used as biological control agents (BCAs)^15,16^. Most BCAs colonize the root surface (rhizosphere), whereas some can also enter the root interior and establish endophytic populations^17^. BCAs protect plants from pathogens via antagonism and/or induction of systemic resistance in host plants^17,18^. BCAs can directly suppress pathogen growth via the production of inhibitory antibiotic chemicals and competition for nutritional resources^14,17–19^. Various antibiotic chemicals, including iron-chelating compounds (siderophores) and antibiotics, have been identified^14,18,19^. In addition, many BCAs have been found to produce and emit microbial volatile organic compounds (mVOCs) that are directly toxic to soil pathogens^19–21^. For instance, several cyanogenic *Pseudomonas* strains have been found to inhibit the tobacco black root rot-causing fungal agent *Thielaviopsis basicola*^22^ and potato late blight-causing oomycete agent *Phytophthora infestans*^23,24^ by producing antifungal mVOCs including the volatile respiratory inhibitor hydrogen cyanide (HCN). So far, more than 1300 mVOCs have been identified, with the major chemical classes being alcohols, ketones, aromatic compounds, terpenes, organic acids, esters, aldehydes, sulfur compounds, alkanes, and nitrogen compounds^19^. Among them, dimethyl disulfide has been the most extensively studied and successfully patented and commercialized as a soil fumigant (Paladin™) in greenhouses and open fields (Paladin Technical US EPA Reg. No. 55050-3)^19,20^.

In soybean, several bacterial and fungal strains have been reported for their biological control effects against *Phytophthora sojae*^25^, *Sclerotinia sclerotiorum*^26,27^, *Fusarium solani*^28^*, Rhizoctonia solani*^28–30^, *Pythium aphanidermatum*^31^, *Phytophthora nicotianae*^32^, and *Sclerotium rolfsii*^33^. Several *Pseudomonas* bacterial strains have been found to produce antifungal mVOCs against *S. sclerotiorum*, a fungal pathogen with a broad host range of over 400 plant species including soybean^26^. Gao et al.^34^ reported that the application of the rhizobium strain, *Bradyrhizobium* sp. BXYD3, or the arbuscular mycorrhizal fungus (AMF), *Glomus mosseae*, from maize roots (*Zea mays* L.) significantly decreased the occurrence and development of RCR in soybean roots. Interestingly, the root exudates of soybean plants inoculated with *Rhizobium* and/or AMF significantly inhibited *C. ilicicola* mycelial growth, suggesting that inoculation with these microbes promotes the production of antibiotic substances in soybean plants^34^.

We have previously reported the isolation of endophytic^35^ and rhizosphere^36,37^ bacteria belonging to diverse genera from different plant species inhabiting Japan. In this study, we evaluated the antifungal activities of these bacterial strains against the fungal pathogen, *C. ilicicola*, and identified three *Pseudomonas* bacterial strains (OFT2, OFT5, and Cab57) with strong antifungal activity, which may aid in the development of BCAs for the effective and eco-friendly management of RCR in soybean production.

## Results

### Identification of bacterial strains with antifungal activity against *C. ilicicola*

Thirteen bacterial strains (11 endophyte and two rhizosphere bacteria) were tested in this study, which were previously isolated from various plant species in different prefectures in Japan (Table 1)^35–37^. All 11 endophytic bacteria, including four *Pseudomonas* strains (OFT2, OFT5, RH6, and RH7), possess a gene encoding 1-aminocyclopropane-1-carboxylate (ACC) deaminase^35^. The two rhizosphere *Pseudomonas* strains Cab57^37^ and Os17^36^ exhibited biocontrol activity against damping-off and root rot caused by *Pythium ultimum* in cucumber plants^36^.

**Table 1:** Bacterial strains used in this study, which were isolated from various plant species in different prefectures (localities) in Japan.

Strains	Species	Inhabiting types	Host plants	Localities	Accession no
Cab57^37^	*Pseudomonas protegens*	Rhizosphere	Shepherd’s purse	Hokkaido	AP014522
Os17^36^	*Pseudomonas* sp.	Rhizosphere	Rice	Ibaraki	AP014627
HA3^35^	*Streptomyces* sp.	Endophyte	Apple (fruit)	Aomori	LC075701
HK1^35^	*Pantoea* sp.	Endophyte	Apple(fruit)	Aomori	LC075700
HK3^35^	*Nocardia* sp.	Endophyte	Apple(fruit)	Aomori	LC075702
MF6^35^	*Streptomyces* sp.	Endophyte	Apple(fruit)	Iwate	LC075711
MF7^35^	*Streptomyces* sp.	Endophyte	Apple(fruit)	Iwate	LC075703
OFT2^35^	*Pseudomonas* sp.	Endophyte	Carrot (root)	Ibaraki	LC075708
OFT5^35^	*Pseudomonas* sp.	Endophyte	Turnip (root)	Ibaraki	LC075709
RH10^35^	*Mycobacterium* sp.	Endophyte	Sweet pepper (fruit)	Mie	LC075704
RH2^35^	*Mycobacterium* sp.	Endophyte	Sweet pepper (fruit)	Mie	LC075705
RH6^35^	*Pseudomonas* sp.	Endophyte	Sweet pepper (fruit)	Mie	LC075706
RH7^3,5^	*Pseudomonas* sp.	Endophyte	Sweet pepper (fruit)	Mie	LC075707

**Figure 1:**
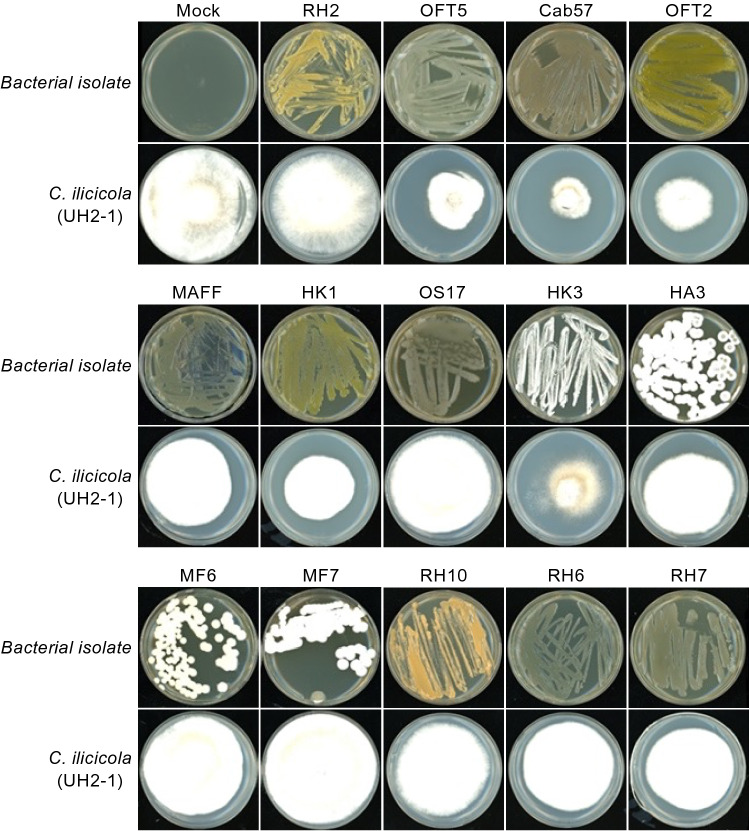
Suppressive effects of endophytic and rhizosphere bacteria on *Calonectria ilicicola* (UH2-1) mycelial growth determined using the double-plate chamber method. The plate images were taken 14 days after incubation. The growth inhibition rates (%) against nine different *C. ilicicola* isolates are shown in the Table 2.

The impact of bacterial VOCs on the mycelial growth of nine different *C. ilicicola* isolates with different pathogenic properties obtained from different prefectures in Japan^10^ was tested using the double-plate chamber method by growing the bacteria and fungi in the same atmosphere, but physically separated from each other, which is the most widely used method for the in vitro assessment of VOC-mediated microbial interactions. The 13 bacterial strains showed significantly different inhibitory effects on *C. ilicicola* growth (Fig. 1; Table 2). Among the 13 bacterial strains tested, two *Pseudomonas* sp. strains (OFT2 and OFT5) and one *Pseudomonas protegens* strain (Cab57) showed particularly high average inhibition rates (≥ 35%) against mycelial growth of all nine *C. ilicicola* isolates: 13–84% and average 46% for OFT2; 17–86% and average 35% for OFT5; and 30–86% and average 47% for Cab57. Interestingly, the growth inhibition effects of the three *Pseudomonas* strains were more evident against the highly virulent *C. ilicicola* isolates, UH2-1, AID1-12, and Y11-1b^10^, with inhibition rates of 57, 58, and 84% by OFT2, 36, 36, and 86% by OFT5, and 50, 53, and 86% by Cab57, respectively (Fig. 1; Table 2). All remaining bacterial strains showed an average inhibition rate of ≤ 31% (Fig. 1; Table 2). Meanwhile, a *Pantoea* sp. strain (HK1) and a *Streptomyces* sp. strain (MF7) showed high inhibition activity against the *C. ilicicola* isolate Y11-1b of 78 and 64%, respectively.

**Table 2:** Antifungal effects of bacterial VOCs on the mycelial growth of nine different *Calonectria ilicicola* isolates in the double-plate chamber test.

Bacterial strains	Inhibition rate (%) of *C. ilicicola* growth									
	UH2-1	A1D1-12	Y11-1b	NI1-3-1	S-1	SN2-1	S-4	S-5	S-6	AVG
**Cab57**	**50.1 ± 15.1**^**ab**^	**52.6 ± 2.9**^**a**^	**85.5 ± 5.4**^**a**^	**43.0 ± 5.2**^**a**^	29.5 ± 5.6^a^	**41.6 ± 2.1**^**ab**^	25.5 ± 5.1^bcd^	**49.7 ± 8.5**^**a**^	**48.3 ± 8.7**^**a**^	**47.3 ± 1.6**^**a**^
OS17	7.5 ± 5.0^de^	**37.8 ± 4.8**^**b**^	**81.9 ± 6.9**^**a**^	14.7 ± 6.0^bcd^	12.2 ± 4.4^bc^	**32.9 ± 11.1**^**bc**^	20.8 ± 2.9^bcde^	26.1 ± 12.0^abc^	16.7 ± 5.7^cde^	27.8 ± 2.7^cd^
HA3	11.8 ± 8.5d^cde^	4.5 ± 4.8^e^	15.3 ± 6.4^d^	− 0.7 ± 1.1^d^	3.2 ± 1.1^c^	21.4 ± 16.2^bcde^	18.2 ± 7.4^bcde^	9.4 ± 10.1^c^	− 2.0 ± 5.3^e^	9.0 ± 2.1^gh^
HK1	**37.6 ± 5.2**^**abc**^	**33.3 ± 4.8**^**bc**^	**77.7 ± 1.4**^**ab**^	**36.2 ± 6.6**^**ab**^	3.2 ± 5.6^c^	3.3 ± 5.8^de^	27.6 ± 9.9^bc^	27.5 ± 10.9^abc^	**36.9 ± 3.0**^**abc**^	**31.5 ± 1.4**^**bc**^
HK3	24.1 ± 13.8^bcde^	0.0 ± 0.0^e^	12.0 ± 2.9^d^	2.0 ± 3.5^cd^	0.0 ± 0.0^c^	− 0.7 ± 1.2^e^	25.5 ± 5.1^bcd^	6.1 ± 5.3^c^	− 0.7 ± 3.1^de^	7.6 ± 1.8^gh^
MF6	0.0 ± 0.0^e^	1.9 ± 1.9^e^	**55.8 ± 7.8**^**b**^**c**	2.7 ± 6.4^cd^	1.3 ± 2.2^c^	20.2 ± 4.2^bcde^	**36.3 ± 5.3**^**ab**^	14.1 ± 8.8^c^	− 3.4 ± 3.1^e^	14.0 ± 1.8^fg^
MF7	5.1 ± 12.3^de^	9.0 ± 2.9^de^	**64.2 ± 3.6**^**abc**^	26.3 ± 10.8^abc^	6.4 ± 4.4^c^	**37.9 ± 2.6**^**ab**^	**47.0 ± 3.0**^**a**^	**51.7 ± 10.3**^**a**^	29.4 ± 18.4^abc^	**30.8 ± 1.7**^**bc**^
**OFT2**	**57.3 ± 1.7**^**a**^	**58.3 ± 5.6**^**a**^	**84.3 ± 5.3**^**a**^	**32.9 ± 11.2**^**ab**^	12.8 ± 1.1^bc^	**59.1 ± 3.8**^**a**^	**32.8 ± 9.2**^**ab**^	**46.3 ± 11.9**^**ab**^	**34.6 ± 3.3**^**abc**^	**46.5 ± 1.4**^**a**^
**OFT5**	**36.2 ± 14.0**^**abc**^	**35.9 ± 11.1**^**b**^	**85.7 ± 4.2**^**a**^	26.2 ± 5.4^abc^	21.8 ± 9.9^ab^	**33.5 ± 3.9**^**bc**^	24.1 ± 7.1^bcde^	16.8 ± 4.2^bc^	**38.9 ± 16.6**^**ab**^	**35.5 ± 4.3**^**b**^
RH10	5.2 ± 3.4^de^	1.9 ± 1.9^e^	15.3 ± 6.4^d^	18.8 ± 2.1^abcd^	5.8 ± 0.0^c^	9.4 ± 10.1^cde^	6.0 ± 3.4^e^	2.7 ± 3.1^c^	− 3.3 ± 1.1^e^	6.9 ± 2.2^h^
RH2	2.9 ± 1.6^e^	3.2 ± 2.9^e^	5.4 ± 8.4^d^	− 2.0 ± 2.0^d^	1.9 ± 3.3^c^	6.1 ± 10.6^fde^	12.7 ± 6.4^cde^	6.0 ± 11.2^c^	− 2.0 ± 2.0^e^	3.8 ± 1.3^h^
RH6	**30.2 ± 12.9**^**bcd**^	26.9 ± 3.8^bc^	**45.5 ± 13.5**^**c**^	22.9 ± 14.6^abcd^	10.3 ± 2.9^bc^	26.1 ± 12.1^bcd^	19.5 ± 0.4^bcde^	6.7 ± 4.1^c^	16.7 ± 5.7^cde^	22.8 ± 1.1^de^
RH7	14.1 ± 4.5^cde^	19.9 ± 7.3^cd^	**45.5 ± 13.5**^**c**^	**36.1 ± 16.1**^**ab**^	5.1 ± 2.2^dc^	26.9 ± 10.1^bcd^	7.3 ± 4.6^de^	2.0 ± 3.5^c^	16.1 ± 6.8^cde^	19.2 ± 4.6^ef^

Different letters indicate significant differences at the 5% level (Tukey’s Honest Significant Difference (HSD) test). Inhibition rates ≥ 30%, and the three *Pseudomonas* strains with high antifungal activity (Cab57, OFT2 and OFT3) are highlighted by bold fonts.

**Figure 2:**
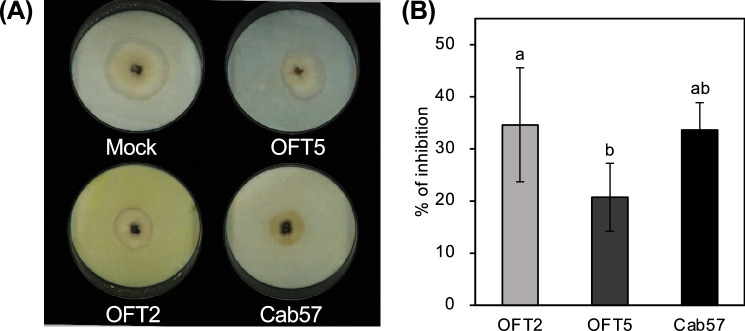
Suppressive effects of the culture supernatants of *Pseudomonas* strains (OFT2, OFT5, and Cab57) on *C. ilicicola* (UH2-1) mycelial growth. The supernatant prepared from the TSA medium without bacterial culture was used as the control (mock). (**A**) Plate images of *C. ilicicola* (UH2-1) growth in the presence of mock and bacterial culture supernatants, (**B**) mean (± standard deviation [SD]) growth inhibition rate (%) relative to the mock control (n = 9). Different letters next to error bars indicate that the means are significantly different from each other as per Tukey’s honest significant difference (HSD) test (p < 0.05).

We prepared bacterial culture supernatants of the three *Pseudomonas* strains (Cab57, OFT2, and OFT5) to examine whether these bacteria also produce and secrete antifungal substance(s) into the surrounding environment using the *C. ilicicola* isolate, UH2-1. The results showed that the culture supernatants of all three bacterial strains significantly inhibited *C. ilicicola* mycelial growth compared with the mock control (Fig. 2).

### Evaluation of the biological control activities of the *Pseudomonas* strains against *C. ilicicola* infection

**Figure 3:**
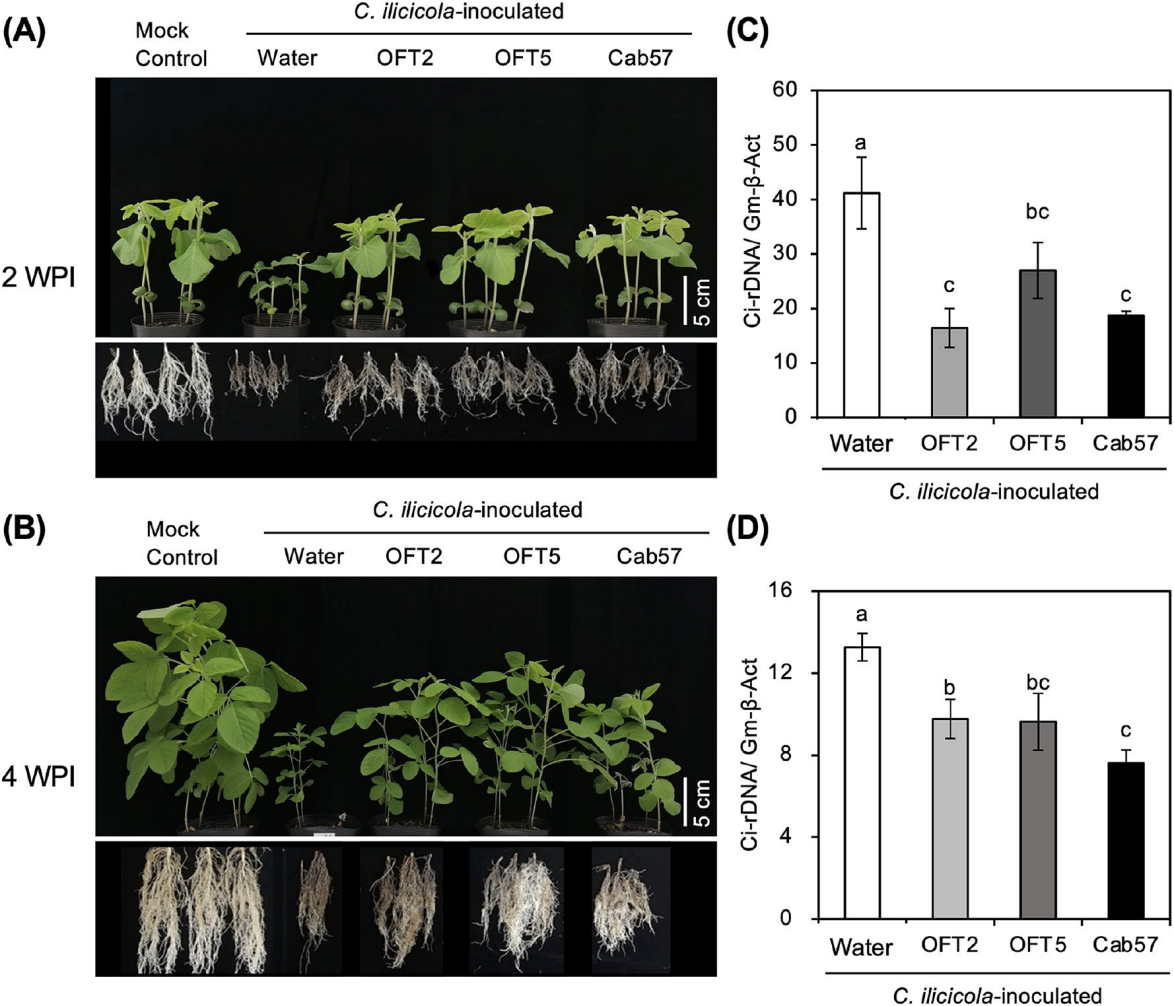
Plant growth features of soybean seedlings (**A**) 2 and (**B**) 4 weeks post-inoculation (WPI) with *C. ilicicola* (UH2-1), and the relative fungal growth (Ci-rDNA/Gm-b-Act) in the roots at (**C**) 2-WPI and (**D**) 4-WPI (n = 20). Different letters next to error bars indicate that the means are significantly different from each other as per Tukey’s HSD test (p < 0.05).

**Figure 4:**
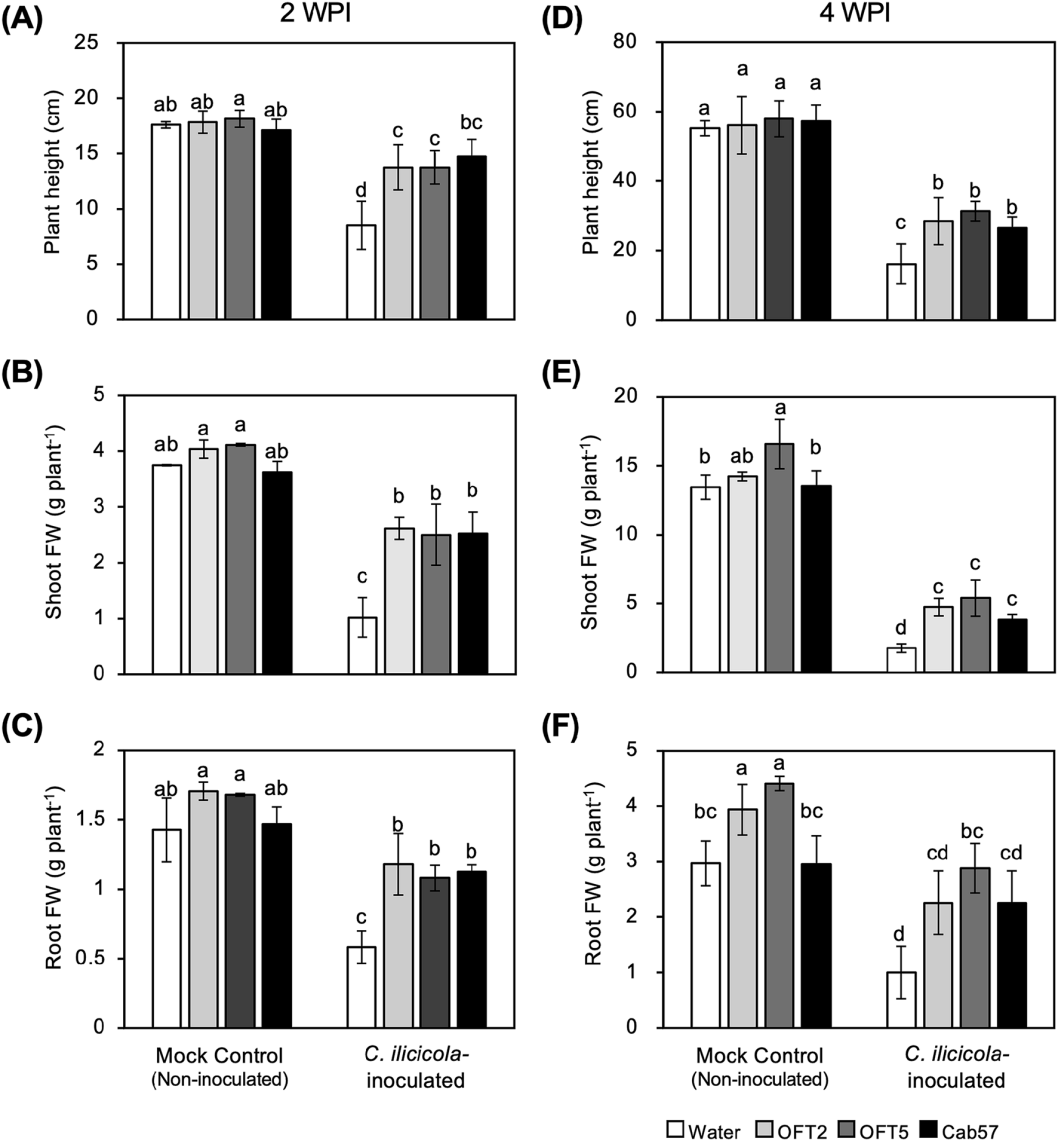
Effects of *Pseudomonas* strains (OFT2, OFT5, and Cab57) on the (**A**,**D**) plant height, (**B**,**E**) shoot fresh weight, and (**C**,**F**) root fresh weight of soybean bean plants inoculated or non-inoculated with *C. ilicicola* (UH2-1) at (**A**–**C**) 2-WPI and (**D**–**F**) 4-WPI (n = 20). Different letters next to error bars indicate that the means are significantly different from each other as per Tukey’s HSD test (p < 0.05).

As the *Pseudomonas* strains, OFT2, OFT5, and Cab57, showed strong antifungal activity against *C. ilicicola* (Figs. 1 and 2; Table 2), we further investigated whether these bacterial strains exhibited any biological control activity in soybean plants against RCR caused by *C. ilicicola* (UH2-1). Compared with the disease-free mock control, a marked reduction in plant growth parameters, including plant height and fresh weight, was observed in *C. ilicicola*-inoculated water control plants at both 2-WPI (Figs. 3A, 4A–C) and 4-WPI (Figs. 3B, 4D–F). In contrast, seed application with OFT2, OFT5, and Cab57 significantly alleviated the negative impact of *C. ilicicola* infection on soybean plant growth at both sampling time points (Figs. 3A,B; 4A–C,D–F), although it could not restore the growth to the levels of the mock control. The negative impact of *C. ilicicola* infection was most drastic in the roots, as shown by the short height and small volume of the roots compared to the water control (Figs. 3A,B, 4C,F). Moreover, seed application of OFT2, OFT5, and Cab57 significantly reduced the root damage, as shown by the significantly recovered root volume and (Fig. 3A,B) fresh weight (Fig. 4C,F).

Consistently, the relative fungal growth of *C. ilicicola* was significantly reduced by the application of the *Pseudomonas* strains when compared with the water treatment (Fig. 3C,D). The highest reduction was observed for OFT2 (60%), followed by Cab57 (54%) and OFT5 (34%) at 2-WPI (Fig. 3C). No significant differences in the reduction of relative fungal growth were observed between the *Pseudomonas* strains. A similar trend was observed at 4-WPI, where the *Pseudomonas* strains reduced the relative fungal growth by 42% for Cab57, 27% for OFT5, and 26% for OFT2 (Fig. 3D).

### Effects of the *Pseudomonas* strains on plant growth

In the control plants (not inoculated with *C. ilicicola*), no negative effects of the *Pseudomonas* strains were observed at either 2 or 4 weeks after seed sowing (Fig. 4). A significant increase in the fresh weights of the roots by OFT2 and the roots and shoots by OFT5 was observed at 4 weeks after seed sowing (Fig. 4E,F). No such effect was observed with Cab57.

## Discussion

Biological control is important as an eco-friendly and practical approach for plant disease management in various crops, particularly for controlling soil-borne pathogens^9,14^. Several bacterial and fungal isolates have been isolated and studied for the biological control of various soybean diseases^25–33^, but no BCAs with practical and commercial potential for *C. ilicicola* control have been reported. Application of rhizobia and/or AMF alleviates RCR severity in soybean roots^38^. However, further investigation is needed on the overall RCR control effect to use these microbes as BCAs, as *C. ilicicola* can also invade the roots via the nodules, which may lead to even more severe RCR symptoms^39^. Moreover, it is technically challenging and expensive to in vitro propagate obligate biotrophs, including AMF, for practical use^40^. The success of this study in identifying the *Pseudomonas* strains (OFT2, OFT5, and Cab57) with strong biological control activity against *C. ilicicola* will aid in the development of effective BCAs to control soybean RCR. These findings may be particularly significant for soybean production in Japan, where RCR is one of the major limiting factors for soybean grain yield^4^.

The *Pseudomonas* sp. OFR2 and OFT5 are endophytic bacteria isolated from carrot and turnip, respectively, and both express ACC deaminase^35^. Inoculation of OFT5 into tomato seedlings can enhance their salt stress tolerance by reducing stress-related ethylene production^41^. *Pseudomonas protegens* Cab57 is isolated from the rhizosphere of Shepherd’s purse^37^, and exerts biocontrol activity against damping-off and root rot caused by *Pythium ultimum* in cucumber plants^36^. In this study, three *Pseudomonas* strains produced and emitted mVOCs with potent antifungal activity against nine different *C. ilicicola* isolates (Fig. 1; Table 2). Each bacterial strain exhibited different growth inhibitory effects on different *C. ilicicola* isolates, and some bacterial strains, HK1 and MF7, showed a high inhibition rate for a particular *C. ilicicola* isolate (Y11-1b) (Table 2). In addition, the three selected *Pseudomonas* strains (OFT2, OFT5, and Cab57) had high growth inhibitory effects against *C. ilicicola* isolates with high virulence (UH2-1, AID1-12, and Y11-1b) in soybean plants^10^ (Fig. 1; Table 2). Whether this biased inhibitory effect on different *C. ilicicola* isolates has any biological significance remains to be clarified. Taken together, these results indicate some genetic and biochemical variations among the *Pseudomonas* and *C. ilicicola* isolates, which determine the outcomes of the interactions between individual bacterial and fungal isolates. The *Pseudomonas* strains (OFT2, OFT5, and Cab57) were also shown to produce and secrete antifungal substance(s) against *C. ilicicola* (UH2-1) (Fig. 2), indicating that these bacteria can suppress *C. ilicicola* growth by producing antifungal mVOCs and secretory metabolites. In support of this notion, genomic analysis of strain Cab57 revealed that it harbors the gene clusters for production of HCN^37^, a potent antifungal mVOC^22–24^, and the antibiotics 2,4-diacetylphloroglucinol, pyrrolnitrin and pyoluteorin etc.^16,37^. It would be important and interesting to assess the role of these chemical compounds in the inhibition activity on *C. ilicicola* growth.

In soil inoculation experiments, *C. ilicicola* inoculation caused severe root rot and growth retardation in the soybean seedlings. In contrast, co-inoculation of *C. ilicicola* with a *Pseudomonas* strains (OFT2, OFT5, or Cab57) significantly reduced *C. ilicicola* proliferation in the roots (Fig. 3C,D) and rescued the plant growth inhibition caused by *C. ilicicola* infection to some extent (Fig. 3A,B; Table 2). These results demonstrate that the *Pseudomonas* strains, OFT2, OFT5, and Cab57, have strong biocontrol activities against *C. ilicicola*, which may be used for the development of BCAs to manage RCR during soybean production. Our results suggest that the mechanism of biocontrol activity of these bacteria is at least partly associated with the antagonistic suppression of *C. ilicicola* growth via the production and release of antifungal mVOCs (Fig. 1; Table 2) and secretory metabolites (Fig. 2). Whether these bacterial strains are also capable of inducing host resistance remains to be determined in future studies.

No negative effects of the *Pseudomonas* strains (OFT2, OFT5, and Cab57) on soybean plant growth were observed (Figs. 3A,B, 4), which is important for their practical use as BCAs in RCR management. Rather, an increase in the fresh weight of the roots by OFT2 and the roots and shoots by OFT5 was observed (Fig. 4E,F). These growth-promoting effects of OFT2 and OFT5 may be attributed to their ACC deaminase activity, as ethylene generally reduces the plant growth^35,41^. On the other hand, the strain Cab57 showed no significant plant growth promoting effect, even though it also contains a homologue of ACC deaminase gene in its genome (PPC_RS20245)^37^. Therefore, further study remains to clarify the contribution of ACC deaminase activity to soybean plant growth.

In summary, we found that three *Pseudomonas* strains, OFT2, OFT5, and Cab57, significantly inhibited the development of soybean RCR caused by the fungal pathogen, *C. ilicicola*. This biological control effect relies on the antagonistic suppression of *C. ilicicola* growth via the production and release of antifungal substances. These bacterial strains may provide a basis for the development of BCAs for the effective management of soybean RCR. However, the specific substance(s) responsible for the suppression of *C. ilicicola* growth and the efficacy of these bacterial strains in controlling RCR in actual soybean fields require further elucidation in future studies.

## Methods

### Plant material and growth conditions

Soyabean (*Glycine max*) cv. Enrei was used for all experiments in this study. Enrei is a *C. ilicicola*-susceptible cultivar popularly cultivated in Hokuriku and Northeast regions of Japan. The seeds of Enrei were obtained from the Institute of Agrobiological Sciences, NARO, Japan. All the experimental procedures including the collection of plant material complied with institutional, national and international guidelines and legislations.

The seeds were pre-conditioned in a moisture-saturated plastic box for 24–48 h at 25 °C. The seeds were then sown in commercially available pre-fertilized and granulated soil (Nippi No.1, Nippon Hiryo, Tokyo, Japan) in 144-cm^2^ plastic pots at a depth of 20 cm. Five seeds were sown per pot (12 cm × 12 cm × 20 cm; 1500 mL) and grown in a greenhouse at 25 °C and 50% relative humidity. All soils used in this study were autoclaved at 120 °C for 1 h one day before seed sowing to eliminate any effects of other soil pathogens.

### *C. ilicicola* culture and inoculation

Fungal mycelia of nine *C. ilicicola* isolates (Table 2) were cultured on potato dextrose agar (PDA) plates at 25 °C for 1–2 weeks or until fungal mycelial growth reached the edges of the Petri plates (9 cm)^2,10^.

The *C. ilicicola* isolate, UH2-1, was used for the inoculation of soybean (Enrei) as described previously^10,42^. Briefly, 5–8 pieces (~ 5-mm cubes) of PDA with vigorously growing *C. ilicicola* mycelia were placed in a 500-mL flask containing 200 g of wheat bran-vermiculite medium (wheat bran/vermiculite/water 1:1:3, w/w/v) and incubated at 26 °C for 10–14 days, until the fungal mycelia fully covered the medium^42^. This culture was used as the inoculum, and an inoculum-soil mixture was prepared by mixing the inoculum with Nippi No.1 soil to generate a concentration of 1% (w/v). The soil mixture was then filled in plastic pots (12 × 12 × 20 cm; 1500 mL), into which five seeds were sown per pot.

### Culture of bacterial strains

The bacteria (Table 1) were cultured overnight on tryptic soy agar (TSA) plates at 28 °C. For biological control assays, bacteria were cultured in tryptic soy broth (TSB) medium with shaking (150 rpm) at 28 °C for 24 h.

### Measurement of antifungal effects of bacterial VOCs

The possible antifungal effects of mVOCs produced by the bacterial strains against *C. ilicicola* were examined using the double-plate chamber method^43,44^. Nine *C. ilicicola* isolates were used in this study (Table 2). Briefly, 10 μL of overnight bacterial culture in TSB broth (OD_600_ = 0.4) was spread on the surface of a TSB agar plate (5.2 cm in diameter) and incubated overnight at 25 °C. On the other hand, a 5-mm diameter mycelial plug of *C. ilicicola* was inoculated individually at the center of the PDA agar plate (5.2 cm in diameter). The TBS broth agar plate with a bacterial strain was placed onto the PDA agar plate inoculated with a *C. ilicicola* isolate so that the two plates faced each other. The contact surfaces of the two plates were sealed with parafilm to obtain a double-plate chamber, and the plates were incubated at 25 °C in the dark for 10–14 days. The average distance between the surfaces of the two plates was 1.5 cm. The fungal growth rate was represented as colony diameter (cm). A double-plate chamber without bacterial strains was used as the control. The experiment was repeated thrice, with four replicates each. The percentage inhibition of fungal growth was calculated as follows:

$${\text{Percentage inhibition}}=\frac{(C-T)}{C} \times 100$$

where C represents the colony diameter (mm) in the mock plate (with water as a control) and T represents the colony diameter (mm) in the bacterial plate.

### Measurement of antifungal effects of bacterial culture supernatant

The bacterial culture supernatants of the three *Pseudomonas* strains (Cab57, OFT2, and OFT5) were investigated for their antifungal activities against *C. ilicicola*. Bacterial culture supernatants were prepared according to the method described by Pethani^45^, with slight modifications. The *Pseudomonas* strains (Cab57, OFT2, and OFT5) were cultured on TSA plates at 28 °C for 24 h. The TSA medium containing the bacterial culture was homogenized by passing through a syringe several times and mixed with an equal volume of sterilized water. The slurry mixture was centrifuged at 10,000×*g* for 60 min, and the supernatant was filtered through a 0.22-μm Millipore filter (Whatman^®^ 9911-1302 Syringe filter) to remove any remaining bacteria. A filter paper (Whatman) was soaked in 4 mL of bacterial supernatant and placed in a petri plate (9-cm diameter). A small agar plug of *C. ilicicola* culture was inoculated onto the filter paper at the center of the plate and incubated at 28 °C for 7 days. The supernatant prepared from the TSA medium without bacterial culture was used as the control (mock).

### Evaluation of the biological control activities of the *Pseudomonas* strains against *C. ilicicola*

The three *Pseudomonas* strains, Cab57, OFT2, and OFT5, were evaluated for their biocontrol activities against *C. ilicicola*. The bacteria were cultured in 30 mL of TSB medium with shaking (150 rpm) at 28 °C for 24 h and collected by centrifuging at 10,000 rpm for 10 min at 4 °C (TOMY MX-301 high-speed refrigerated microcentrifuge). The resultant bacterial pellets were washed twice via resuspension in sterile water, and the density was adjusted to 0.4 at OD_600_ (approximately 10^7^ cells mL^−1^) in sterile water.

After seed sowing, as described in section “Plant material and growth conditions”, 1 mL of the bacterial suspension was poured concentrically around each seed, and the top of the pot was covered with a 2-mm layer of autoclaved pre-fertilized peaty soil Supermix-A (Sakata Seed Corporation, Yokohama, Japan). The pots were arranged in a completely randomized design with four replicates in a greenhouse maintained at 25 °C and 50% relative humidity. Pot positions were randomly changed daily to minimize positional effects in the greenhouse, and plant density and size were small enough to induce mutual shading among different plants. Two and four weeks post-inoculation (WPI), plant growth parameters, including plant height and shoot and root fresh weights, were recorded.

### Real-time quantitative polymerase chain reaction (qPCR) for examination of relative fungal growth

Relative fungal growth of *C. ilicicola* (UH2-1) was detected using qPCR, as described previously^10^. Briefly, genomic DNA was extracted from the whole root system using a MagExtractor (Toyobo, Osaka, Japan), following the manufacturer’s instructions. Three root samples were represented for each replicate, and there were four replicates for each treatment and three biological replicates (n = 36). Real-time qPCR was performed on a Thermal Cycler Dice TP800 system (Takara Bio. Inc., Otsu, Japan) using SYBR premix Ex Taq mixture (Takara) with cycles of 95 °C for 5 s, 55 °C for 20 s, and 72 °C for 20 s. Relative fungal growth was expressed as *C. ilicicola* rDNA amplification fold-relative to host *β-actin* gene amplification. The PCR primers used were (1) primers targeting the intergenic spacer region of the *C. ilicicola* rDNA: CiIGSF (forward) = 5′-TCCATTGCCTCTATTTATCCTGC-3′ and CiIGSR (reverse) = 5′-GCGTAAAGATTTTCCAACCCG-3′^46^; (2) primers for soybean β-actin gene 11 (Glyma.15G050200): Gm-β-ActinF (forward) = 5′-GAGCTATGAATTGCCTGATGG-3′) and Gm-β-ActinR (reverse) = 5′-CGTTTCATGAATTCCAGTAGC-3′.

### Experimental design and data analysis

Antifungal assays were performed in three independent replicates, each consisting of three culture plates. Biological control assays were performed using three independent biological replicates, each consisting of four pots with five plants per pot for each treatment. All experiments were performed twice and representative data from one experiment are shown.

The mean values were compared using Tukey’s honest significant difference test (p < 0.05) with XLSTAT Version 2017 (Addinsoft).

## Data Availability

The complete or partial genome sequences of 13 bacteria used in the present study are available in the DDBJ/EMBL/GenBank database under accession numbers as indicated in Table 1.
